# Exploring what is reasonable: uncovering moral reasoning of vascular surgeons in daily practice

**DOI:** 10.1186/s12910-022-00881-x

**Published:** 2023-01-09

**Authors:** Kaja Heidenreich, Mia Svantesson, Marit Karlsson, Anders Bremer

**Affiliations:** 1 Faculty of Medicine and Health, University Health Care Research Centre, Örebro University, S-Huset, 2nd Floor, 70182 Örebro, Sweden; 2 Department of Health, Medicine and Caring Sciences, Linköping University, 58183 Linköping, Sweden; 3 Faculty of Health and Life Sciences, Linnaeus University, 35195 Växjö, Sweden

**Keywords:** Decision-making, Medical ethics, Physicians, Qualitative research, Surgeons

## Abstract

**Background:**

Vascular surgery offers a range of treatments to relieve pain and ulcerations, and to prevent sudden death by rupture of blood vessels. The surgical procedures involve risk of injury and harm, which increases with age and frailty leading to complex decision-making processes that raise ethical questions. However, how vascular surgeons negotiate these questions is scarcely studied. The aim was therefore to explore vascular surgeons’ moral reasoning of what ought to be done for the patient.

**Methods:**

Qualitative, semi-structured interviews were conducted with 19 vascular surgeons working at three Swedish university hospitals. Data were analysed according to systematic text condensation.

**Results:**

The surgeons’ moral reasoning about what ought to be done comprised a quest to relieve suffering and avoid harm by exploring what is reasonable to do for the patient. Exploring reasonableness included to shift one´s perspective from the vessels to the whole person, to balance patient’s conflicting needs and to place responsibility for right decision on one´s shoulders. The shift from blood vessels to the whole person implied gaining holistic knowledge in pondering of what is best, struggling with one´s authority for surgery through dialogue, and building relationship for mutual security. To balance patient’s conflicting needs implied weighing the patient’s independence and a sense of being whole against ease of suffering, respecting the patient’s will against protecting life and well-being, and weighing longer life against protecting the present well-being. Finally, to place responsibility on one´s shoulders was conveyed as an urge to remind oneself of the risk of complications, withholding one’s power of proficiency, and managing time during the illness course.

**Conclusions:**

This study contributes to uncovering how moral reasoning is embodied in the vascular surgeons’ everyday clinical discourse as a tangible part of their patient care. The results underpin the significance of moral considerations in the assemblage of medical knowledge and technical skills to further understand vascular surgeons’ clinical practice. The clinical application of these results is the need of forums with sufficient possibilities for articulating these important moral considerations in everyday care.

**Supplementary Information:**

The online version contains supplementary material available at 10.1186/s12910-022-00881-x.

## Introduction

Moral reasoning concerns the exploration of what is right and wrong or virtuous and vicious, and through moral reasoning one argues about what ought to be done [1]. In a clinical health care setting, moral reasoning aims to reveal well-supported arguments for judgments, decisions and actions [2, 3]. However, to know what ought to be done is a challenge and in determining the right action for the individual patient, health care professionals negotiate contextual, social, clinical, ethical and personal concerns in decision-making [4, 5].

Moral reasoning as an empirical phenomenon has traditionally interested moral psychologists who have explored people’s moral reasoning as a cognitive capacity, which develops during childhood and could be measured empirically [6]. Kohlberg’s well-known studies of children’s moral development described how children develop their thinking about right actions from self-interest to thinking in terms of what is socially desirable to the last and highest level of thinking based on applying ethical rules and principles [7].

However, trying to uncover how health care professionals deal with ethical issues is methodologically complex, partly because the term ‘ethical issues’ lacks a clear definition [8–10]. Literature in ethics further displays broad and varied concepts aiming to capture dealing with ethical issues ranging from considerations [11], perspectives [12, 13], views [14, 15], conceptions [16], moral values [17], ethical reasoning [18, 19], justifications [20], and decision-making [21, 22]. Even if there are considerable differences between these concepts, they also point to the intricate methodological problems of trying to capture the dealing with ethical issues in the clinical context of patients and clinicians.

The present study employed the phenomenon moral reasoning in decision-making to explore how surgeons deal with ethical issues in everyday care. In the applied qualitative methodology, the phenomenon was defined implicitly to be sensitive to the practical, social and medical context [23]. Moral reasoning in decision-making is situated in a specific context producing meaning to answer the moral question about what ought to be done for the patient. This meant not strictly separating facts and values, but rather seeing them as intertwined to explore the phenomena from the perspective of the clinicians [24, 25].

The setting of the present study is vascular surgery, which offers a range of treatments to relieve burdensome symptoms of pain and ulcers and to prevent sudden death by rupture of blood vessels [26]. The surgical procedures involve risk of injury and harm, which increases with age and frailty leading to complex decision-making processes [27, 28]. Additionally, questions regarding the equity in the delivery of vascular surgery in different parts of Sweden has emerged [29]. According to the Swedish vascular registry, the number of vascular procedures differ substantially between regions. These differences are not considered to be due to differences in patients’ needs, but are perhaps instead influenced by the surgeons’ moral reasoning about what ought to be done for the patient [29].

Ethics of general surgery have been described at a theoretical level [30–32], but empirical research about surgeons’ moral reasoning in the decision-making process is scant. Two studies described general surgeons’ ethical dilemmas of deciding the right treatment and their reasoning [33, 34]. A qualitative meta-aggregation has examined the issue of informed consent for surgery from the perspective of patients´ and surgeons [35] and a questionnaire has examined trauma surgeons’ ethical issues showing difficulties related to communication and autonomy [36]. Vascular surgeons’ attitudes to hypothetical scenarios have been described [37, 38] as well as brief descriptions of ethical difficulties in acute vascular care [39–41], but research related to patient care in practice is lacking.

Thus, there is a need to explore vascular surgeons’ moral reasoning within their daily practice in order to understand how ethical issues are handled and how moral reasoning is displayed in decision-making. Therefore, the aim of this study was to explore vascular surgeons’ moral reasoning of what ought to be done the patient.

## Methods

The study adopted an explorative and interpretative design within a qualitative methodology utilizing semi-structured interviews [42].

### Participants

The seven largest clinics for vascular surgery at university hospitals, according to the Swedish vascular registry [43], were invited by email to the head of the department for participation in the study. Three clinics agreed, and the surgeons at these clinics received written and verbal information about participating in the interviews. An information meeting about the study aim, methods and practicalities of the research project was held at each clinic by the first author attended by the potential participants. Nineteen surgeons agreed to participate. At the first two clinics, all (*n* = 14) surgeons agreed to participate. At the third clinic, a consecutive sampling strategy to recruit five participants was applied. For a description of the demographic data of the participants, see Table 1.

**Table 1:** Demographic characteristics of the participants (*n* = 19)

Gender, *n* (%)	
Male	14 (73)
Female	5 (28)
Age, mean (range)	48 (35–69)
Years of experience, mean (range)	
In vascular surgery	15 (3–37)
Since graduation	21 (7–43)
Participants, *n*	
Hospital 1	8
Hospital 2	6
Hospital 3	5

### Data collection

The face-to-face semi-structured interviews by the first author were held in an office at the end of the participants regular working day in the out-patient clinic. The interviews focused on patients the participants had met the same day, in contrast to general expereinces of difficulties. The opening of the interview was “I would like to talk with you about patients you have met today. Are there any of them where you experienced difficulties or uncertainty about the further handling or had doubts about what was the best option for the patient? Could you please tell me about them?” Probes were posed in order to further explore their reasoning, like “Can you describe more…”, “What do you mean…”, and “Please elaborate on…”. Follow-up questions were further adapted to the situation and could comprise factual circumstances, alternatives for actions or perceived expectations, reactions and preferences of the patient and their relatives. The surgeons reasoned in total about 39 (mean 2, range 1–6) patients. The interviews were audio-recorded, lasted on average 37 min (range 18–66 min) and transcribed verbatim by a research secretary.

### Data analysis

The interviews were analysed according to systematic text condensation [44], facilitated by the software program, NVivo-11 [45]. The interviews were listened through for accuracy in transcription of the medical language and was read repeatedly to identify preliminary themes. The text was then coded by identifying and sorting sentences and paragraphs containing information about the phenomenon and the aim of the study. The phenomenon was deliberately defined openly to avoid a too early narrowing of what could be understood as moral reasoning. Analysing the participants’ clinical reasoning was a prerequisite to further analyse and understand their moral reasoning. The coding was strictly inductive starting from the surgeons’ stories about their patients and the reasoning concerning what they ought to do. Medical and more factual information in their reasoning necessary for answering the moral question of what ought to be done was included in the analysis as a part of the phenomenon.

With the growing number of codes, those sharing similarities were assembled into coding groups according to content or preliminary themes. Codes and coding groups were continuously validated against the data, moving between the parts and the whole to refine and sort the codes into groups and corresponding subgroups. The findings were synthesized through the condensation, interpretation and reformulation of the themes and sub-themes in a continual process of co-assessment by the authors.

The project was conducted in accordance with the declaration of Helsinki and received ethics approval from the Swedish Ethical Review Authority (No. 2019-04387). All participants gave written, informed consent prior to the interview. The study followed the COREQ checklist for the reporting of qualitative research [46] (Additional file 1).

## Results

The moral reasoning of the vascular surgeons encompassed an exploration of what is reasonable in a quest for relieving suffering and avoiding harming the patient. This implied to shift one´s perspective from the vessels to the whole person, to balance patient’s conflicting needs and to place responsibility for right decision on one´s shoulders. The themes include nine sub-themes, see Table 2.

**Table 2:** Surgeons’ moral reasoning described by main theme, themes and sub-themes

Main theme	Exploring reasonableness questing for relieving suffer and avoiding harm		
Theme	To shift one´s perspective from vessels to the whole person	To balance patient’s conflicting needs	To place responsibility for right decision on one´s shoulders
Sub-theme	Gaining holistic knowledge in the pondering of what is best	Weighing independence and sense of being whole against ease of suffering	Reminding oneself of the risk of complications
	Struggling with authority through dialogue	Respecting patient’s will versus protecting life and well-being	Withholding one’s power of proficiency in decision-making
	Building a relationship for mutual security	Weighing longer life against protecting present well-being	Managing time during the illness course

### To shift one´s perspective from vessels to the whole person

In the surgeons’ exploration of reasonableness, their moral reasoning started with the blood vessels, but shifted to gaining holistic knowledge in the pondering of what is best, reasoning about struggling with authority for surgery through dialogue and the engendering of mutual security through their relationship.

**Gaining holistic knowledge in the pondering of what is best **implied gaining knowledge about the patient’s vascular problem as well as their health to form considerations about what ought to be done. Patient’s discomfort caused by the vascular problem and how this affected their life was a dominating focus. Surgeons contemplated about trying to understand the loss of function in daily life and the magnitude of suffering. A particular focus was patient’s experiences of pain and how this affected movement, sleep and daily activities as well as the need for pain medication.

Despite gathering information from the patient’s records and x-ray results, meeting face-to-face was a prerequisite to gain valid information about the patient’s broader health. To notice body language, movement, voice and meet patient’s eyes was expressed to give important knowledge about strength and frailty. This informed the surgeon of how much surgical trauma the patient could endure and thereby what kind of technical solution they should choose. Considerations of the patient´s life expectancy were important in choosing between different surgical techniques. For instance, endovascular procedures were viewed as gentler for frail patients, but had less durability in relieving suffering long time. Open techniques implied higher risk of complications, but with longer durability.

“How he is, just the short walk from the waiting room, does he get dyspnoeic or can he walk fast? Does he have pain while walking, when he takes his shoes off? Will he be able to lie on the operation table, what would be the risks in this?” (Surgeon 9, hospital 2)

**Struggling with authority through dialogue** implied that both secure authority and ambivalent authority was conveyed about what ought to be done. Secure authority emerged as an assertiveness of what was in the patient’s best interest. The procedure was viewed to be of benefit for the patient, associated with justified risk of complications and according to the patient’s views and wishes conveyed through their dialogue. The reasoning could concern how old and frail patients struggled with complicated medical information. The patients often asked the surgeons what they ought to do, and their responses reflected what they considered to be in the patient’s best interest. Secure authority could be conveyed as long as they did not experience substantial doubts about the benefit for the patient or the procedure was associated with serious risk of complications.

“He left that to me. It is quite common that the patients say: ‘You can decide,’ or ‘Do what you think’ or ‘What do you think?’ We invite or try to invite the patient to have their own opinion and see if they want, some do, and other just want to be taken care of.” (Surgeon 10, hospital 2)

In decisions about prophylactic measures, high-risk surgery, or procedures with a scarce knowledge base, the dialogue with the patient was especially important for authority. The surgeons reasoned over the importance of the patient understanding the risk of injuries and complications in the surgeons’ experience of justified authority. They did not share the decision-making responsibility with the patient, but the dialogue grounded their experiences of providing warranted authority prior to performing complicated procedures.

… a procedure I exposed them for may cause complications. Then it´s very important to have met them, spoken to them, looked them in the eyes. That they have understood the risks, that they go into it with one’s eyes open. (Surgeon 19, hospital 3)

Furthermore, being unable to reason with patients about what ought to be done seemed to further weaken secure authority. Surgeons struggled to reach old and frail patients not understanding complex medical information or lacking decision-making capacity. It could be about patients with dementia who had need of vascular procedures to avoid amputation, but lacked capacity to participate in the decision-making process and the procedure. Being on the operation table could be like torture for a confused patient and, in these situations, no authority could be justified in advance and reasoning over abstaining procedures occurred. To fulfil the patient’s needs, an amputation could be the most reasonable option to ease suffering.

**Building a relationship for mutual security** implied establishing security for the patient as well as for the surgeon prior to surgical measures. The patient was described as being in a difficult situation of suffering and loss of health and the relationship established in the outpatient clinic should engender security in a vulnerable situation.

“You show that you’re a human being, that you’re not just a doctor and that you understand their concerns, that you try to establish a relationship, that you show yourself as a decent person, that you display knowledge, so they can believe you have sufficient experience to make a decision for them.” (Surgeon 14, hospital 3)

Security could be conveyed by an endeavour to give clear-cut information about the low risk of rupture and the benefit of many years more to live for patients with minor aortic aneurysm. Continuity was important for security, particularly regarding high-risk procedures, both when operating and completing the follow-up in the outpatient clinic. Surgeons also expressed a need for security for themselves, especially prior to performing risky procedures. This security emerged from the relationship and dialogue with the patient as well as their own conviction that the surgery was justifiable.

There are something personal I think, that we should know each other, you should know your doctor, know who you´ve spoken to, not just meet different doctors. Someone you can meet after the operation. (Surgeon 1, hospital 1)

### To balance patient’s conflicting needs

The moral reasoning revealed how the conflicting needs of the patient corresponded with important values in the decision-making process. Values at stake included independence and a sense of being whole, ease of suffering, protecting life and well-being, length of life, and respecting the patient’s will. These values emerged in opposition and the surgeons tried to achieve a reasonable balance on behalf of the patient.

**Weighing independence and sense of being whole against ease of suffering** concerned patients with critical ischemia described as being in a crucial situation. An endeavour to avoid amputation was expressed. The patients were often old and frail with limited muscular strength for using a prosthesis after an amputation, and losing a leg would mean a loss of ability to move independently and being more dependent on support from others. The necessity of an amputation was experienced as being loaded for both the surgeon and the patient, as losing a leg also implied being mutilated with a concomitant loss of a sense of being whole.

“It’s about being mutilated, to lose a part of the body. You’re born with your parts and you want to be buried with the same parts.” (Surgeon 3, hospital 1)

Independence and sense of being whole was balanced against ease of suffering, as critical ischemia implied chronic leg ulcers with substantial suffering from severe pain, bad odours, painful dressings and troublesome side effects. The surgeons described trying to relieve the patients’ suffering by performing surgical procedures and preventing the disease from deteriorating into a life-threatening condition.

That an amputation can become a relief from pain, from serious infections. (Surgeon 6, hospital 1)

**Respecting the patient’s will versus protecting life and well-being** signified a conflict between the patient’s wishes for surgical care and the surgeons’ desire to protect the patient’s life and well-being when patients declined amputation of their severely impaired leg. They expressed accepting the patient’s decision, but struggled with the consequences for the patient’s life and well-being. Critical ischemia could be life threatening, and an amputation could protect the patient’s life and, by relieving severe pain, be the turning point for improved well-being, however, at the cost of threatening independence and a sense of being whole.

I said that I think the best for you concerning how your foot looks, the suffering you´re describing och our limited possibilities to influence this…the best is probably that we think about taking away the foot. He did not lash out, but he said; No, I don´t want that, it´s too early. (Surgeon 13, hospital 3)

Surgeons arranged for new appointments with the patient in the outpatient clinic as well as talking to the next-of-kin to try to influence the patient. Questions from previous patients about why they waited so long to carry out the procedure could sometimes help a doubtful patient to make a decision that the surgeon thought was in the patient’s best interest. However, surgeons expressed a conviction of both respecting the patient’s right to decline care and the need for justified authority for surgery.

“Even if his position was to keep the leg when he left, I think it was a meaningful visit for him, his reactions were adequate and he explained why he reasoned as he did.”(Surgeon 12, hospital 3)

**Weighing longer life against protecting present well-being** was found in the reasoning about prophylactic surgery for aneurysms. Surgical procedures could prevent future death from rupturing vessels, but pose a risk of serious complications that could threaten the patient’s present well-being and demanded balancing. An assessment of the risk of rupture was based on professional recommendations concerning indication for surgery. The indication represented a recommendation about when surgical treatment could be considered, but the surgeons’ further reasoning concerned whether the prevention of sudden death should be performed for the particular patient.

On one hand quite low risks with the operation, in the longer perspective expected good survival and untreated, substantial risk of rupture. Thera are other patients that are much more difficult. (Surgeon 13, hospital 3)

Surgeons deliberated on whether prophylactic treatment was meaningful in the perspective of the patient’s life expectancy and health. For patients of high biological age with chronic diseases and limited health, their reasoning tilted in favour of protecting present well-being and abstaining from surgical treatment and a longer life. For patients with perceived good health and function, the balancing tilted in favour of a longer life and surgery. Aspects of technical complicity were considered, and struggle was described when the perceived risk of rupture was comparable to the risk of the surgical procedure. To justify a prophylactic procedure, the benefit of the procedure had to overweigh the risks.

“Why we abstain treatment is because the costs for the patient, the risks and the suffering of the patient, is too large. So it’s not on the basis of economic resources or something.” (Surgeon 8, hospital 2)

### To place responsibility for right decision on one´s shoulders

The surgeons deliberated recurrently over the demanding responsibility they placed on their shoulders. Reasoning concerned reminding oneself of the risk of complications and carefully achieve a reasonable balance between risk and benefit. Responsibility also conveyed withholding their power of proficiency in decision-making and responsible manage of time in following the patient during their illness course.

**Reminding oneself of the risk of complications** by well-meant surgical procedures implied reflections upon how they approached the patient regarding risks. A surgical procedure should not only succeed technically, but should also be accomplished without the patient being affected by complications influencing their health and life. Through assessing the complexity of the technical procedure, they tried to consider the risk of adverse outcomes and whether the procedure could be considered justifiable. Being open about risks of complications was a responsibility implying striving to inform the patient in a balanced way by neither neglecting important risk nor frightening the patient into refusing a procedure. The patients were experienced as struggling with the information and what that meant for them.

“It’s a quite difficult case. There are technical aspects and risks to consider. You do not want to put the patient in a worse situation. Sometimes it’s most right to do nothing.” (Surgeon 7, hospital 2)

**Withholding one’s power of proficiency** in decision-making meant that the surgeons’ power as decision-makers implied a corresponding responsibility to always exercise this power for the benefit of the patient. Vascular surgery was described as a highly technical discipline, demanding advanced practical skills, which should always be utilized in the patient’s best interest. However, advanced procedures could also be an attractive technical challenge for the surgeon, which could blur the ability to make responsible judgments for patients. That an operation could be performed did not necessarily imply that it ought to be performed, and the responsibility was to always consider the patient’s best interests in the bigger picture of health and life for decisions to be justifiable.

“It turns out to be a more technical challenge to solve this aneurysm than an actual benefit for the patient. This is high-technological work. But what I sometimes miss is that sure, we can do this, we can solve this by a large operation or a series of complicated procedures, but this is an 87-year-old man with general declining health, what is his quality of life today and what will this measure lead to?” (Surgeon 12, hospital 3)

The surgeons also had to restrain their power to avoid overriding the patient’s voice and wishes. They deliberated over the challenge of offering the patient surgery, while simultaneously being sensitive the patient’s authentic wishes. Restraining power was difficult when old and frail patients explicitly relinquished the decision-making to the surgeon.

I could have a preconceived idea about what the patient wants, but they actually wants something different. Then I could start convincing the patient about something they actually don´t want and they might not be in able to question or say no because there are power and hierarchy in the room. If you have cognitive problems or have had a stroke and struggle with understanding, it could be tricky what´s right. (Surgeon 16, hospital 3)

**Managing time during the illness course** meant slowing down as well as hastening the decision-making process. Surgeons tried carefully to judge the urgency of the surgical procedure they thought was in the best interest of the patient. On one hand they tried to give the patient sufficient time to make a decision, and, on the other hand, not enduring too much suffering and loss of general health during the process. A carefully hastening of the decision-making process was needed when encountering patients suffering from critical ischemia where a surgical procedure could avoid worsening or where an amputation would be in the patient’s best interest. The surgeons tried to capture whether the patient was ready for making a decision about surgery or needed more time to experience security prior to surgery. This was especially important for risky procedures and prophylactic surgery. The surgeon could also need more time when they felt uncertainty, often by scheduling for new appointments in the outpatient clinic.

“If you know this won’t turn out good, you shouldn’t wait too long either because meanwhile the patient is immobilized because of this withered leg under a longer period, the patient becomes generally declined with tougher rehabilitation and difficulty walking with a prosthesis.” (Surgeon 4, hospital 1)

## Discussion

**Fig. 1:**
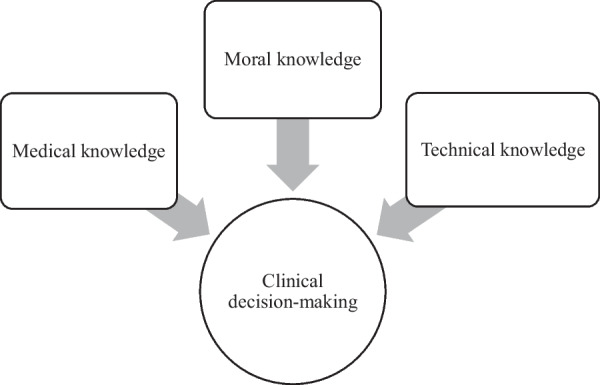
Sources of knowledge for clinical decision-making in vascular surgery

The moral reasoning of the vascular surgeons encompassed an exploration of what is reasonable in the quest to relieve suffering and avoid harming the patient. This exploration comprised to shift one´s perspective from the blood vessels to the whole person, to balance patient’s conflicting needs through handling value conflicts and to place responsibility for right decision on one´s shoulders with considerations over own moral character. We argue that the surgeons, through their moral reasoning, acquire *moral knowledge,* which they use in addition to their medical knowledge based on numerical data as well as technical knowledge of surgical possibilities to make good clinical decisions for patients (Fig. 1).

First, some consideration about the results in light of ethical theory. The surgeons had substantial value conflicts which they strived to balance to move forward in their decision-making process. Value conflicts are well-describe in the literature of medical ethics through The Four Principles approach and has both impact and relevance for clinicians [2, 47]. The balancing act between the patient’s conflicting needs corresponds to the midlevel principles of respecting patient autonomy, and promoting beneficence and non-maleficence. The broad principle of beneficence could be seen as some kind of, however controversial, moral goal of healthcare [48]. Several of the values the vascular surgeons weighted corresponded to the principle of beneficence, but were actually opposing each other. Weighing a longer life against protecting the patient’s present well-being from deliberations concerning prophylactic surgery both concerned beneficence as well as avoid harming the patient. Weighing the patient’s independence and a sense of being whole against the ease of suffering concerning critical ischemia and amputation also have correspondence with this broad principle. The four principles, which meritoriously capture important areas of ethics in health care, have lesser efficacy in actually guiding actions and solving conflicts between values and principles and are hence in need of concretization in clinical decision-making [49]. However, even if the moral reasoning of the surgeons has some correspondence with the four principles, the role or function of the moral reasoning in the surgeons’ decision-making process is ambiguous. The further discussion explores whether the surgeons’ moral reasoning could be understood as a way the surgeons gather knowledge; *moral knowledge* to answer the question of what ought to be done for the patient in addition to their *medical knowledge*.

Central to the surgeons’ moral reasoning was their relationship with the patient. To answer what ought to be done, the surgeon and the patient together explored the patient’s needs and wishes for care, the degree and meaning of the suffering for the patient, and the patient’s perceptions of surgical risk. Additionally, the surgeons explored the patients’ general health to judge what surgical trauma the patient could endure and the life perspective under which the surgical treatment would be meaningful. This reasoning could be interpreted as an acquirement of *moral knowledge* from the relationship with the patient to inform the clinical decision. Walker describes in her moral epistemology what moral knowledge is, where to look for it, and when to know you have found some. She argues that moral knowledge is located in the human social life and is defined as “a socially embodied medium of mutual understandings and negotiation between people over their responsibility for things open to human care and responses” [50]. Essential to this template is moral responsibility and how people negotiate “who gets to do what to whom and who is supposed to do what for whom” [50]. The surgeons’ exploration of reasonableness could be understood as a gathering of relational, situated and patient-unique moral knowledge from the encounter with the patient. This knowledge is utilized by the surgeons, together with their medical knowledge based on numerical data on groups of patients, as well as their technical skills about surgical measures, to answer the question of what ought to be done for the patient (Fig. 1).

One could argue that the surgeons also obtained strictly objective or medical information from the patient encounter, and that this information answered the moral question about what ought to be done for the patient. However, the gathering of this information was done in a relational and dialogical sphere where information to the moral question was far more than the patient´s health status. The need for meeting the patient prior to a decision was self-evident for the surgeons and could be understood as a need for gaining moral knowledge in the unique patient encounter and, through the dialogue, engender security for both parties. The surgeons justified the warranted authority for surgery through dialogue and reciprocity in the decision-making process, which could be interpreted as a way of balancing the unequal distribution of power between the parties. According to Walker, moral knowledge is neither theoretical nor objective, but something gained according to who we are, how we understand ourselves, and where we come from [50]. Moral knowledge is gained in real time and spaces, culturally situated, and effected through social positions where hierarchical power-relations are the rule. The surgeons deliberated over the hierarchical structure of the patient relationship and their power of proficiency as decision-makers. If not handled with care and responsibility, this power could blur the vision of the surgeons and become an obstacle to achieving responsible decisions with the aim of ensuring that the patient received good care.

Shared-decision making (SDM) has evolved as a key model for decision-making in preference-sensitive decisions and the stories told by the surgeons often uncovered clinical scenarios the surgeons experienced as sensitive to the patient’s preferences [51]. The concept of SDM emphasizes that professionals share possible options with pros and cons with the patient and further elaborate the patient’s opinions and values prior to a decision [52]. Decisions identified as preference-sensitive, equipoise and decisions where patient commitment is necessary for implementation, are appropriate for SDM [53]. The surgeons reasoning on how the dialogue justified their authority to fulfill a surgical measure might be reasoning over the decisional phase described in SDM [52]. However, even if the surgeons emphasized the relationship and participation of the patient in the process, the data gives no support for the surgeons sharing the decisional responsibility with the patient. The surgeons still had to judge a surgical measure as right and responsible to undertake it and their reasoning concerned struggle with authority for frail patients as well as for complex procedures. The withholding of one's own exercise of power was a part of the surgeons’ reasoning over their moral responsibility and was something they reflected over and might be an important prerequisite to elaborate the patient’s views and values. However, the surgeons’ struggled with patients with limited capacity for participation in the decision-making process and poor general health is a described barrier to SDM [54]. The degree of SDM in vascular surgery has been studied empirically in the Netherlands. Audio recorded consultations were judged according to the Option-5 instrument showing low degree of SDM mainly due to insufficient support to the patient in deliberating there options [55, 56].

The surgeon’s moral reasoning explored in this study probably influenced their decision-making, and diversity of moral reasoning could be one explanation for why the number of vascular procedures, according to registry data, differ substantially between regions in Sweden [29]. What is explored as reasonable, what conclusion could be drawn from the encounter and the patient relationship, how value conflicts are balanced, and how power of proficiency is withheld could account for some of these observed differences in vascular procedures [29]. Making clinical decisions and doing vascular surgery is far more than the application of medical science and clinical guidelines and implies a moral and interpretative enterprise that needs to be articulated in discussions of good as well as equal health care.

### Methodological considerations

Three out of seven invited hospitals agreed to participate and this may have adversely affected the validity of the study and reduced the transferability of the results. The declining clinics gave two reasons for not participating: lack of time and interest (*n* = 3); and research ethics concerns about sensitive patient information (*n* = 1). Other reasons that have not been specified could be unfamiliarity with the methods, and concerns about being scrutinized. It could be argued that the participating surgeons might have had a greater awareness of ethical issues in daily practice compared to vascular surgeons in general. The participating clinics might also have a climate of permissive dialogue. However, at two of the clinics, all surgeons participated and no surgeons declined an interview, implying that not just those who are morally sensitive were included. Including three different participating clinics also provided the possibility to capture social diversity, which strengthens the validity of the results. The decision to interview the surgeons about patients they had just met on the same day generated valuable data, which we believe prevented the risk of recall bias and obtaining socially desirable answers. However, the approach to interview the surgeons about real patients and not asking general questions regarding experienced ethical issues lacked the possibility to capture structural prerequisites in work influencing caring for patients, as lack of time, and reasoning concerning acute patients.

## Conclusion

Knowing what ought to be done for the patient is far more than the application of guidelines and utilizing medical knowledge. The moral reasoning of the vascular surgeons reveals how ethics are a tangible part of surgeons’ daily care of patients, embedded in their clinical discourse. During their reasoning of what ought to be done, surgeons explore reasonableness, striving to relieve suffering and avoiding harm. Doing vascular surgery demands moral knowledge, in addition to medical and technical knowledge, which, in these empirical data, was embodied in the assemblage of the patient relationship, value conflicts and the moral character of the vascular surgeons. The clinical application of this knowledge is the need of forums for articulating moral considerations in everyday care as well as the fostering of moral character.

## Supplementary Information

**Additional file 1:** COREQ Checklist.

## Data Availability

The datasets generated and analysed during the current study are not publicly available due current Swedish ethical legislation and European union GDPR act, but are available from the corresponding author on reasonable request, if appropriate permits are obtained from adequate authorities.
